# Substitute for Quinine

**Published:** 1871-02

**Authors:** 


### SUBSTITUTE FOR QUININE.

It is stated, in the *Lancet*, that M. Pavia, an Italian professor of chemistry, has procured an alkaloid from the leaves and roots of boxwood, which he calls bussine. In the experience of several Italian physicians, this substance has been found to possess virtues nearly equal to those of quinine in the treatment of miasmatic fevers. In severel cases, gastric uneasiness, pyrosis, thirst, nausea, giddiness, and tinnitus aurium, were attributed to the use of the remedy.—*N. O. Journal of Medicine.*

ON THE USE OF BROMIDE OF LITHIUM.—Dr. S. Weir Mitchell, in experimenting on the various bromides, finds that the bromide of lithium produces the characteristic action more promptly than any of the other preparations. This salt is very deliquescent and should be given in solution. It contains nearly 92 per cent. of bromine, while the salt of potassium has but 66 per cent., and that of sodium only 72 per cent.

This new salt seems to act efficiently in some cases of epilepsy where the bromide of potassium has failed. It is efficient in lesser doses than the latter salt, and as an hopnotic it is superior to all the other bromides.

He gives a brief history of its use in several cases of epilepsy, where, after treatment with other bromine salts, this was substituted with the effect to produce more profound effect in smaller doses; in one or two cases it seems to have had some control over the disease where the other salts had entirely failed.

Bromide of lithium is less unpleasant than that of potassium, but more so than that of sodium; it is at present much more expensive than either, but if the consumption of it was great it could be furnished at a much lower rate.—*American Journal Medical Science.*

